# Global tropical cyclone extreme wave height climatology

**DOI:** 10.1038/s41598-024-54691-9

**Published:** 2024-02-20

**Authors:** Guisela Grossmann-Matheson, Ian R. Young, Alberto Meucci, Jose-Henrique Alves

**Affiliations:** 1 Department of Infrastructure Engineering, University of Melbourne, Melbourne, Australia; 2 Weather Program Office, Ocean and Atmospheric Research, NOAA, Silver Spring, MD USA

**Keywords:** Physical oceanography, Engineering

## Abstract

A global study of extreme value (1 in 100-year return period) tropical cyclone generated waves is conducted across all tropical cyclone basins. The study uses a 1000 year tropical cyclone synthetic track database to force a validated parametric wave model. The resulting distributions of extreme significant wave height show that values in the North Atlantic and Western Pacific basins are the largest globally. This is partly due to the relative intensities and frequencies of occurrence of storms in these basins but also because the typical velocities of forward movement of storms are larger and hence can sustain the generation of larger waves. These larger values of velocity of forward movement tend to occur at higher latitudes. As a result, in both of these basins the largest extreme waves occur at higher latitudes than the maximum tropical cyclone winds. In all other tropical cyclone basins, storms tend to propagate more east–west and hence the maximum values of extreme significant wave height and wind speed occur at comparable latitudes.

## Introduction

In tropical and sub-tropical regions, tropical cyclones (or hurricanes or typhoons) represent the most extreme meteorological forcing events, generating wind speeds in excess of 50 m/s^1–4^ and ocean significant wave heights in excess of 12 m^5,6^. As such, tropical cyclones have important societal impacts, resulting in damage to coastal infrastructure^7^, coastal flooding^8^ and beach erosion^9^. Compared to higher latitude storms (extra-tropical cyclones), tropical cyclones are characterized by a relatively small well-formed core with an eye of typical radius from 10 to 40 km^2,10,11^ and an asymmetric vortex wind field. The spatial extent of typical tropical cyclones (TCs), as measured by the radius to gales is from 300 to 500 km^12,13^. Waves generated by TCs propagate away from the intense wind regions of such storms as swell and hence impact much larger regions than the intense winds^14–17^.

In recent decades, a range of studies have investigated wave fields within TCs^4,15,18–24^. Despite the complex wind fields of TCs which have a calm eye, strong wind field gradients and rapidly changing wind directions, wave fields are remarkably well defined^5,25^. The wave height field is asymmetric with the maximum significant wave height to the right (northern hemisphere) of the propagating TC. The waves tend to radiate out from the intense wind speed regions near the eye of the TC and appear ahead of the TC as swell. The wave spectrum in the intense wind regions is very similar in form and scaling to those observed in much simpler fetch-limited growth situations^5,22^. Ahead of the storm, the swell and wind sea remain linked through non-linear processes, meaning that the relationships between significant wave height, peak wave period and wind speed scale in a similar manner to fetch-limited cases. The similarity between waves generated in the complex wind fields of TCs and in simpler fetch-limited cases has been attributed to the dominant role played by non-linear wave-wave interactions in both cases^5,6,22^.

The important role played by non-linear interactions in TC generated waves means that it has been possible to develop parametric models which are computationally highly efficient and can reproduce the wave field with acceptable accuracy for engineering and design purposes^6,15–17,23^. Such applications commonly focus on the determination of extreme significant wave height statistics for particular locations. These are typically represented by the 1 in *n* year event (e.g. $n =$ 100-year significant wave height, $H_{s}^{100}$). Extreme value analysis (EVA) is typically used to estimate quantities such as $H_{s}^{100}$. This involves the fitting of an appropriate extreme value probability distribution to recorded or modelled data and the extrapolation to the required probability level ($P_{r}$)^26,27^ (here, for the 100-year event, $P_{r} = 0.01 = 1/100$). The extrapolation of the probability distribution is required if the recorded time series has a shorter duration than the desired return period. The use of synthetic datasets longer than the return period, allows direct estimation without extrapolation^28^.

Over the last two decades, global EVA estimates of extreme significant wave height using hindcast or reanalysis datasets^29,30^, altimeters records^31–34^, from atmosphere and wave model ensembles^28,35^ or based on spatial ensemble data^36^ have been applied. These global analyses do not, however, have the required spatial resolution to determine extreme values within TCs^34,37^, although methods to reduce the impact of such issues have been applied^38,39^.

The present analysis provides, for the first time, global estimates for all TC basins at a resolution capable of defining $H_{s}^{100}$ across each basin. Our analysis identifies the important variables in defining extreme significant wave height in each basin and how these differ between basins. As such, the analysis is aimed at enhancing our understanding of the global extreme wave climate generated by TCs and the potential impacts on society and infrastructure.

To achieve the desired outcomes, a computationally efficient, validated parametric model of TC significant wave height^23^ was used to generate the spatial distribution of significant wave height during the passage of TCs. Initially, recorded TC tracks over the last 40 years were used^40^. However, this approach was found to be unsatisfactory, as the number of TCs at specific locations was inadequate to generate stable extreme value statistics. As a result, a synthetic TC track database, based on recorded TC track statistics, was utilized^41^. From this database, 1000 years of synthetic TCs for each TC basin were selected and the parametric wave model^23^ was used to generate the resulting wave fields. The TC significant wave height database, generated in this manner, allows Direct Return Estimates (DRE)^28^ of $H_{s}^{100}$ defined on a spatial grid for each TC basin.

## Results

### Global distribution of extreme significant wave height across Tropical Cyclone basins

The 100-year return period estimates of significant wave height ($H_{s}^{100}$) were determined using the approach described above for each TC basin. In summary, 1000 years of synthetic TC tracks and wind field parameters were obtained from the STORM database^41^ for each basin. The parametric TC significant wave height model (PModel)^23^ was then used to generate the resulting wave field during the life of each synthetic TC. The three-hourly values of $H_{s}$ from the ensemble of all synthetic TCs were used to determine annual maximum (AM) values on a regular 1.0° × 1.0° grid over each basin. As the duration of the dataset (1000 years) is longer than the desired probability of occurrence (100-years), it was possible to determine $H_{s}^{100}$ at each grid location using the DRE approach (see details in “Methods” section).

Note that the STORM database^41^ has a total duration of 10,000 years. The dataset is, however, stationary, being based on the recorded IBTrACS data^40^. Bloemendaal et al.^41^ provided detailed validation of the first 1000 years of the STORM database. Therefore, this same subset of the full database was selected for the present application. As the dataset is stationary, the selected time window will have no practical impact on the resulting extreme value statistics.

**Figure 1:**
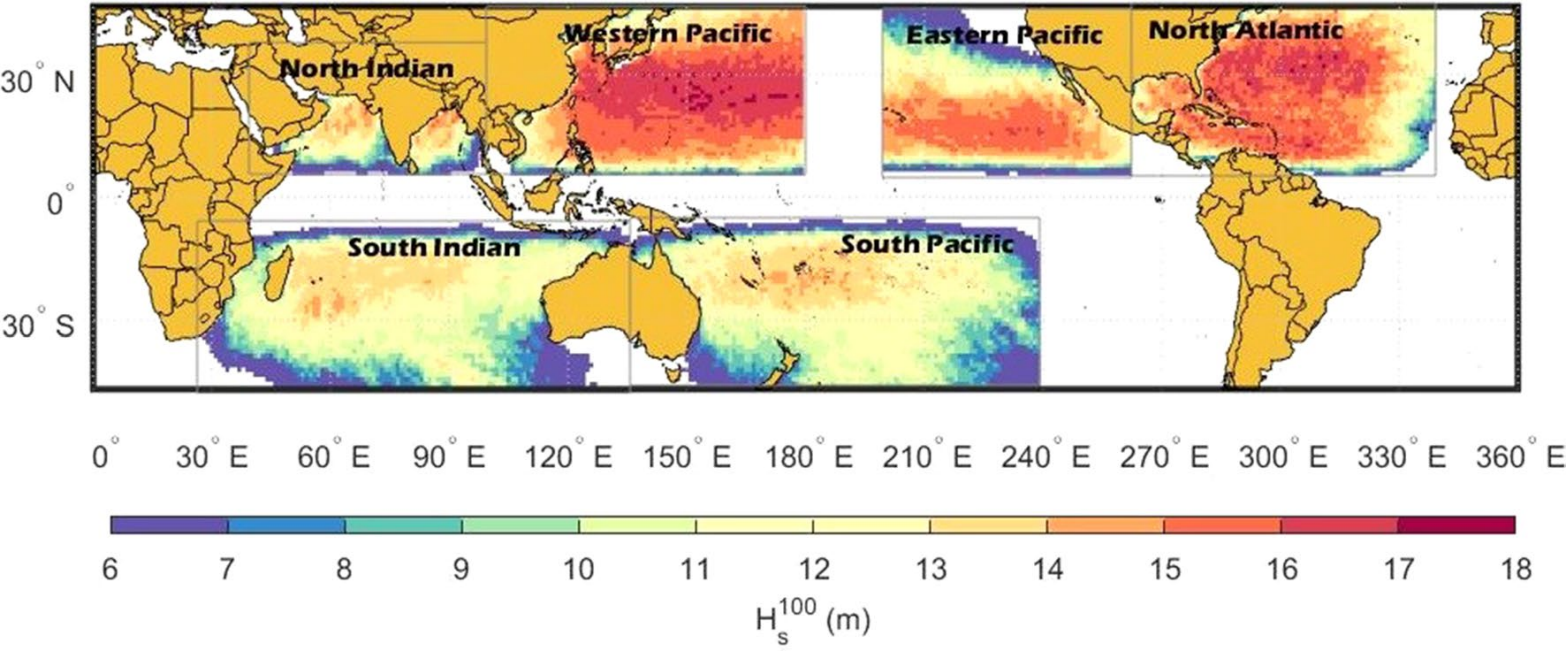
Estimates of 100-year return period significant wave height ($H_{s}^{100}$) under TC conditions for each global TC basin (figure created with Matlab R2023a—mathworks.com).

The six TC basins considered are shown in Table 1—Western Pacific (WP), Eastern Pacific (EP), North Atlantic (NA), North Indian (NI), South Indian (SI) and South Pacific (SP). Although the basic structure of the TC wind field is assumed to be the same across each of these basins, the tracks, frequency of storms and distribution of TC wind field parameters will differ. This will result in different extreme value significant wave height statistics for each basin. Figure 1 shows colour shaded values of $H_{s}^{100}$ across each of the six basins generated using the approach described above. Table 2 shows extreme statistics for each basin, including: maximum values of TC wind speed, $V_{max}$, and significant wave height, $H_{s}^{\max }$ over the 1000 years of TC simulations and the maximum 100-year significant wave height, $H_{s}^{100} (\max )$.

**Table 1:** Boundaries of tropical cyclone basins domains used.

Tropical cyclone basin	
Name	Domain (Excluding land areas)
Western Pacific	5°–45° N, 105°–180° E
Eastern Pacific	5°–45° N, 200°–265° E
North Atlantic	5°–45° N, 265°–340° E
North Indian	5°–45° N, 45°–100° E
South Indian	5°–45° S, 30°–135° E
South Pacific	5°–45° S, 135°–240° E

**Table 2:** Maximum values of TC wind speed, $V_{max}$, and calculated significant wave height, $H_{s}^{\max }$ of the 1000 years of TC simulations and the maximum 100-year significant wave height, $H_{s}^{100} (\max )$ for each tropical cyclone basin.

Tropical cyclone basin	$V_{max}$ (ms^-1^)	$H_{s}^{\max }$ (m)	$H_{s}^{100} (\max )$(m)
	over 1000-year period		
Western Pacific	72.2	19.8	17.4
Eastern Pacific	76.9	19.3	16.4
North Atlantic	78.0	19.9	17.2
North Indian	82.8	18.1	15.5
South Indian	75.2	18.5	14.6
South Pacific	87.6	18.5	14.7

Figure 1 shows that the northern hemisphere (NH) has larger values of $H_{s}^{100}$ than the southern hemisphere (SH), with the maximum value reaching 17.4 m in the Western Pacific (WP) while the highest value of $H_{s}^{100}$ in the southern hemisphere is 14.7 m in the South Pacific (SP) basin.

The North Atlantic (NA) and Western Pacific (WP) basins have similar values of $H_{s}^{100} (\max )$, with 17.4 m for the WP and 17.2 m for the NA. The other NH basins show lower values with $H_{s}^{100} (\max )$ for the Eastern Pacific (EP) of 16.4 m and the North Indian (NI) basin of 15.5 m, respectively. These lower values largely reflect the smaller values of velocity of forward movement of storms in the EP and NI basins compared to NA and WP basins in the STORM database^41^. These smaller values are associated with storms at lower latitudes. Although the values of $H_{s}^{100} (\max )$ in the NA and WP are similar, it is clear from Fig. 1 that there is a larger spatial spread of these large values for the WP basin than for the NA basin.

For the southern hemisphere (SH), the $H_{s}^{100} (\max )$ estimated for the South Pacific (SP) basin was 14.7 m, similar in magnitude to the SI basin at 14.6 m. The maximum values over the full 1000-year synthetic dataset for wind speed, $V_{max}$, are 87.6 m/s for the SP basin and 82.8 m/s for the NI basin. In contrast, the maximum values of significant wave height are 19.8 m for the WP basin and 19.9 m for the NA basic (Table 2). This occurs since the significant wave height is a function of TC wind field variables such as radius of maximum winds, $R_{max}$ and velocity of forward movement, $V_{fm}$, in addition to the maximum wind speed, $V_{max}$^5,6,15,23^.

The results shown in Fig. 1 are consistent with recorded climatology of TCs, with the most intense tropical cyclones occurring in the NH. Data also indicates that 70% of all TCs occur in the NH^3,42^.

### Regional analysis of tropical cyclone extreme waves

Extreme value studies of wave height in TC conditions have typically focused on individual point locations where either relatively long buoy records exist or, for which, high resolution wave modelling has been undertaken. There are almost no studies that show the spatial distribution across basins and none, to the best of our knowledge, that compare different basins. Below, the distribution of $H_{s}^{100}$ across each of the global TC basins is considered and the differences are explained.

For most engineering and coastal planning activities, attention is focused on nearshore extremes, rather than the broader-scale basin climatology as described above. The parametric model used for the present application is a deepwater model (see “Limitations” section) and, as such, does not consider finite depth effects and the fetch limitations resulting from the proximity to shorelines. Therefore, our analysis is not focused on nearshore extremes. Nevertheless, validation of the model itself^23^, and of this analysis (see Validation section), shows good comparisons with buoys within 100 km of coastlines. Therefore, in the analysis of each basin below we do make comments on, not only the maximum extreme significant wave heights in each basin, but whether extreme waves occur relatively close to shorelines.

Numerous studies^5,6,15,23^ have shown that the maximum significant wave height generated within a tropical cyclone, $H_{s}^{\max }$ is not simply a function of the maximum wind velocity, $V_{max}$. In addition, it is a function of the radius of maximum winds, $R_{max}$, the velocity of forward movement of the TC, $V_{fm}$ and, to some extent, the radius to gales, $R_{34}$. This can result in a so-called “extended fetch” within TCs where the waves generated and the storm move forward at comparable speeds. The dependence on these various wind field parameters is captured within the parametric wave model, PModel^23^ (see “Methods” section). In short, TCs with intense winds will not generate extreme significant wave heights if they move slowly ($V_{fm}$ is low), as the waves “outrun” the storm and hence do not stay within the intense wind region for a sufficient time to become very large.

**Figure 2:**
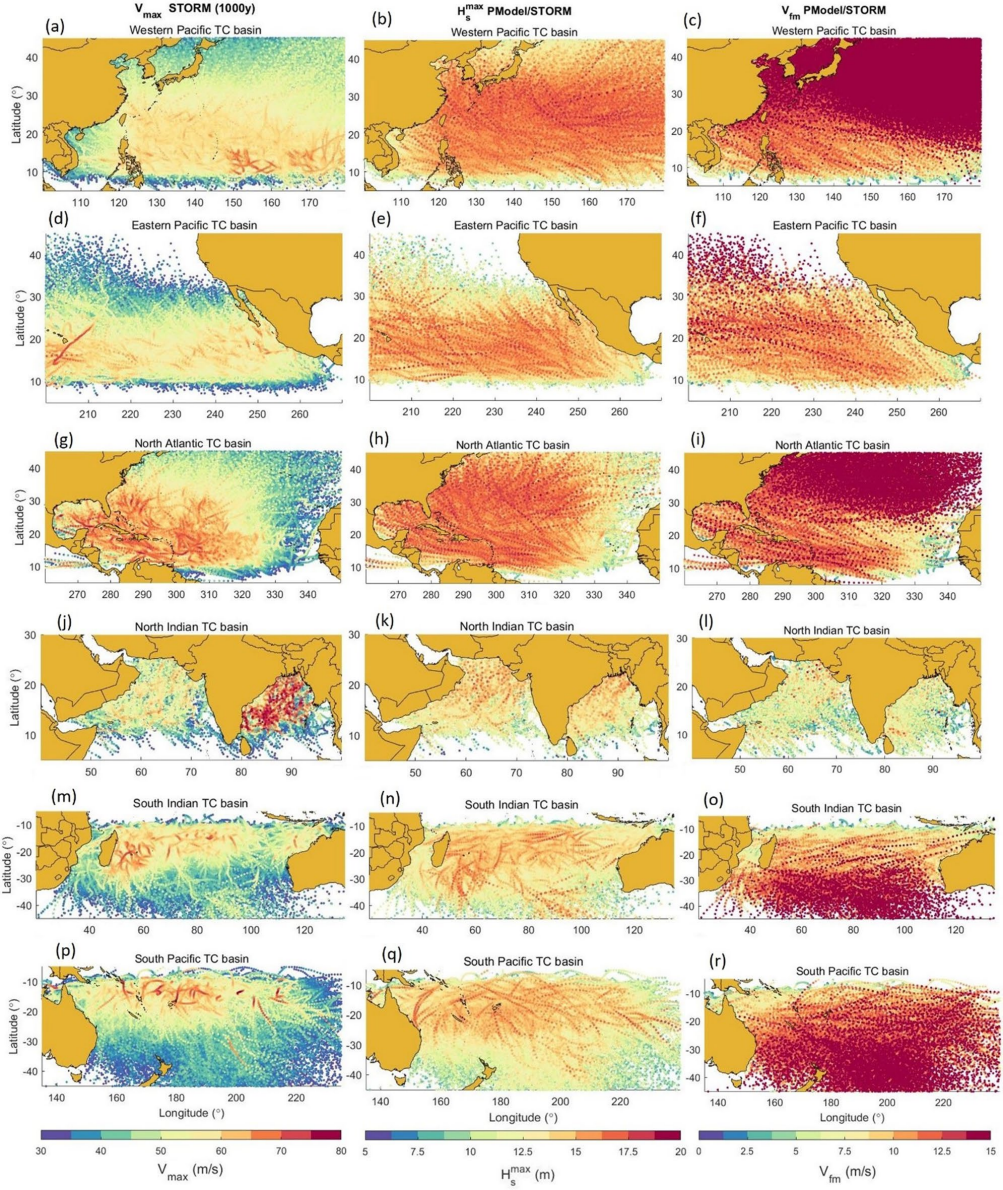
Synthetic tropical cyclone tracks with values of maximum wind velocity, $V_{max}$ (left column), maximum significant wave height, $H_{s}^{\max }$ (middle column) and velocity of forward movement, $V_{fm}$ (right column) (figure created with Matlab R2023a—mathworks.com).

Figure 2 shows plots of the parameters $V_{max}$, $H_{s}^{\max }$ and $V_{fm}$ along each of tracks of the storms within the simulated database. Each of six TC basins is shown. An examination of these results clearly shows that for the WP and NA basins, the largest values of $H_{s}^{\max }$ occur at higher latitudes than the largest values of $V_{max}$. In these basins, tracks tend to have a significant north–south component (recurvature), and as storms propagate to higher latitudes, $V_{fm}$ increases which results in larger “extended fetches” and hence larger waves. This feature is not as clear for WP, SI or SP basins, where tracks tend to have a more east–west direction and hence the largest wind speeds and wave heights occur at similar latitudes. These features are discussed in more detail below in terms of $H_{s}^{100}$ for each TC basin.

Waves in TCs are also impacted by the size of the TC, as measured by $R_{max}$ and $R_{34}$. As storms become larger the effective fetch length increases and the wind-field curvature decreases. This results in larger waves, however, the effect is not as significant as the “extended fetch” and, hence, $V_{fm}$ tends to play a greater role in determining $H_{s}^{\max }$ than $R_{max}$ (or $R_{34}$). However, as shown in “Methods” section, the storms do become larger at higher latitudes, particularly in the WP and NA basins.

### Western Pacific TC basin

**Figure 3:**
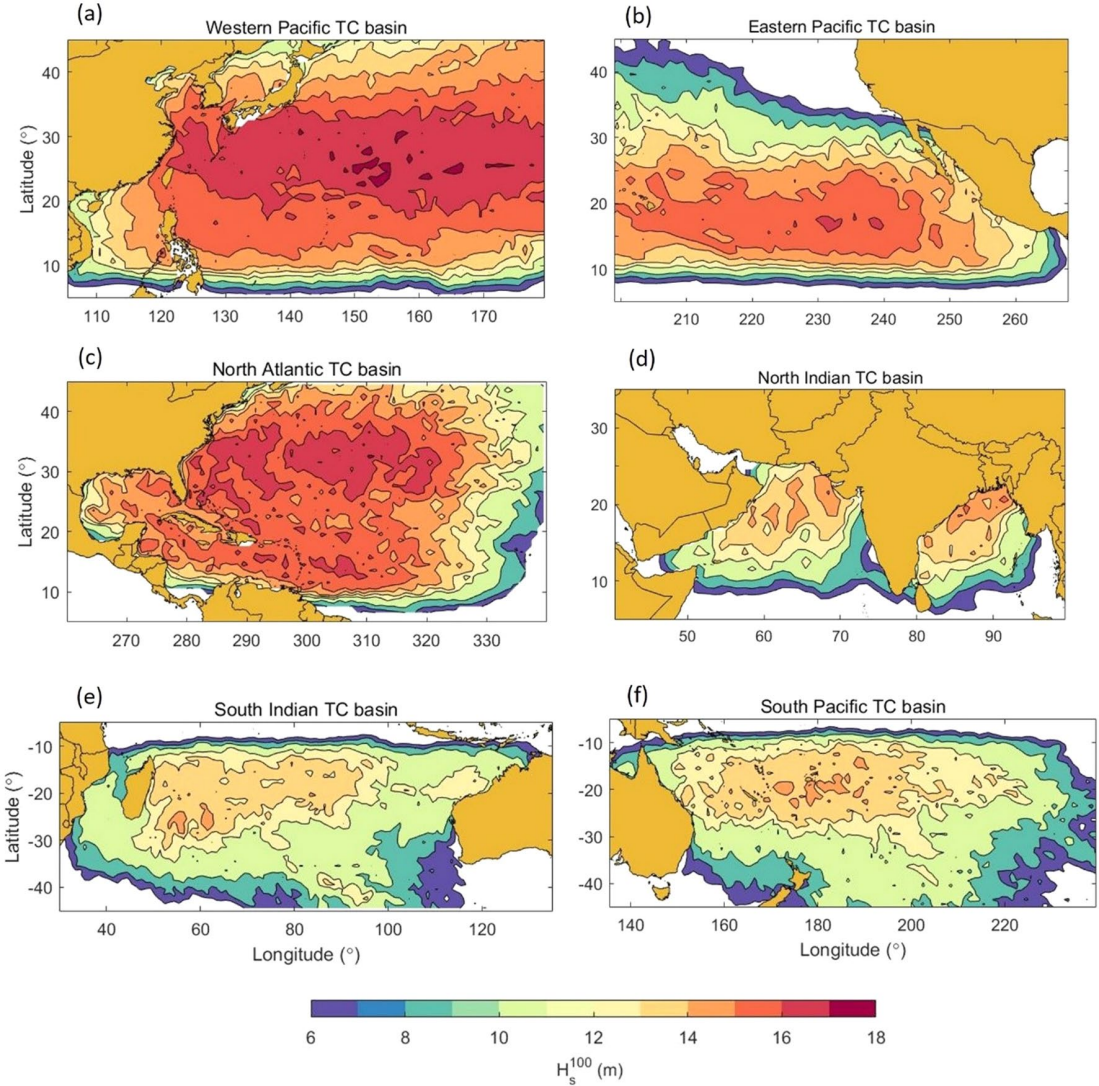
Values of the 100-year return period significant wave height for each tropical cyclone basin (figure created with Matlab R2023a—mathworks.com).

The Western Pacific tropical cyclone basin (WP) contains the South and East China Seas and Sea of Japan in the west and the meridian of 180° to the East. The largest $H_{s}^{100}$ calculated was 17.4 m (Table 2) at 25° N; 152° E. The spatial distribution of $H_{s}^{100}$ is shown as a colour-filled contour plot in Fig. 3a and is characterized by a zone of $H_{s}^{100} >$ 16 m extending in the meridional direction from the East China Sea and between latitudes 20° N–35° N. In the Sea of Japan, $H_{s}^{100}$ reaches a maximum value of 14.6 m, while in the South China Sea values up to 15 m occur. In comparison to the other TC basins, values of $H_{s}^{100} >$ 16 m also occur relatively close to the shorelines of southern Japan and China (Fig. 2a), with clear implications for coastal engineering and shoreline management.

Figure 2a shows that storms with the largest values of $V_{max}$ predominately occur in the latitude band from 10° to 25° N, further south than the band of largest $H_{s}^{100}$. As noted above, this point highlights the fact that for TCs, $H_{s}^{\max }$ is not simply a function of $V_{max}$. This is confirmed by the tracks showing $H_{s}^{\max }$(Fig. 2b), which correspond to the latitudes with the maximum values of $H_{s}^{100}$. Figure 2c shows the track values of $V_{fm}$, showing that these values increase at higher latitudes. As noted above, large values of $H_{s}$ are generally associated with longer wave period and hence higher wave propagation speed^6,22^. As a result, to generate extreme values of $H_{s}$, relatively large values of $V_{fm}$ are required. As a result, the maximum values of $H_{s}^{100}$ occur further north than the largest values of $V_{max}$, where the values of $V_{fm}$ can sustain the growth of such waves.

It should be noted that the STORM database^41^ uses observation data from IBTrACS^40^ that $R_{max}$ is generally a minimum when the central pressure of the storm is at a minimum (largest $V_{max}$). This means that $R_{max}$ will, on average, also increase slightly as $V_{max}$ decreases at higher latitudes, also enhancing wave growth at high latitudes.

### Eastern Pacific TC basin

The Eastern Pacific tropical cyclone basin (EP) is bounded by the southwest coast of the United States and west coast of Mexico in the east, and the western limit is set by the meridian of 200°E (Figs. 2d–f, 3b). The largest value of $H_{s}^{100}$ calculated was 16.4 m (Table 1) at 17° N; 235.5° E. For this TC basin, values of $H_{s}^{100} >$ 15 m occur within a broad longitudinal area between 200° and 245° E and bounded by 13°–25° N in latitude (Fig. 3b).

As seen in Fig. 2d–f, storms in this basin tend to track in an approximately east–west direction. In contrast to the WP basin, they propagate over a much narrower band of latitudes. As a result, as seen in Fig. 2f, values of $V_{fm}$ do not vary significantly and hence, the band of largest $H_{s}^{\max }$, $H_{s}^{100}$ and $V_{max}$ all correspond (13°–25° N). Values of $H_{s}^{100}$ decrease in value near the coast, apparently as a result of few intense storms actually making landfall (Fig. 2d).

In contrast to the WP basin, maximum values of $H_{s}^{100}$ are slightly smaller (16.4 m verses 17.4 m). This is a result of a reduced frequency of storms (14.5/year verses 22.5/year)^41^ but similar values of $V_{max}$ (see Table 2; Fig. 2). Importantly, for the EP basin, the latitudes of storm tracks tend to be further south, where $V_{fm}$ is smaller (compare Fig. 2c and f), resulting in smaller $H_{s}^{100}$.

### North Atlantic TC basin

The North Atlantic tropical cyclone basin (NA) includes the Gulf of Mexico (GoM), the Caribbean Sea and the Atlantic Ocean bounded by the 340° E meridian (Fig. 2g,h,i). The largest value of $H_{s}^{100}$ calculated was 17.2 m (Table 1) at 32° S; 310° E. In contrast to other basins, the NA shows values of $H_{s}^{100} >$ 15 m over a broad range of latitudes (10°–40° N) and above 16 m in the latitude band between 25° and 35° N. This broad distribution is consistent with the tracks of hurricanes in the NA basin which tend to propagate from east to west before turning north along the US coast. The Caribbean Sea shows $H_{s}^{100}$ values reaching 15.5 m associated with the frequent occurrence of TC tracks in this area. The GoM shows a reduction of $H_{s}^{100}$ compared to the Atlantic and Caribbean Sea with values typically around 14 m. As the parametric model does not account for any reduction in $H_{s}$ as a result of the proximity of land, this reduction in $H_{s}^{100}$ is a result of the reduced frequency of occurrence and intensity of GoM hurricanes compared to the North Atlantic coast of the US (Fig. 2g).

The NA has a much lower frequency of occurrence of storms (10.8/year) compared to the WP (22.5/year)^41^, although they tend to be more intense (see Table 2; Fig. 2a,g), with slightly smaller values of $R_{max}$. Importantly, values of $V_{fm}$ are smaller for the NA than the WP, particularly at higher latitudes (Fig. 2c,i). As a result, even though storms in the NA tend to have higher values of $V_{max}$, $H_{s}^{100}$ is slightly lower than the WP (Table 2; Fig. 3a,c).

### North Indian TC basin

The North Indian tropical cyclone basin (NI) includes the Arabian Sea and Bay of Bengal with Asia to the north, the Arabian Peninsula in the west, South-east Asia in the east and is bounded by the Indian Ocean at latitude 5° N to the south (Fig. 2j–l). The largest values of $H_{s}^{100}$ occur in the northern region of the Bay of Bengal with a maximum value of 15.5 m (Table 1) at 20° N; 86° E and a region with $H_{s}^{100} >$ 14 m. The western portion of the NI basin also has some areas with $H_{s}^{100} >$ 14 m, although the majority of the northern Arabian Sea shows values of $H_{s}^{100}$ between 13 and 14 m.

The values of $H_{s}^{100}$ for the NI basin are significantly smaller than both the NA and WP basins. This is consistent with the generally smaller values of $V_{max}$^41^ (although there are some very intense storms, Fig. 2j), the significantly smaller values of $V_{fm}$ (Fig. 2l) and a much lower frequency of occurrence of storms (2.0/year)^41^ than either the NA or WP basins.

### South Indian TC basin

The South Indian tropical cyclone basin (SI) is bounded by latitudes of 5° S to the north and 40° S to the south with Africa in the west and Australia to the east (Fig. 2m–o). The largest value of $H_{s}^{100}$ calculated for this basin was 14.6 m (Table 1) at 25° S; 55° E. This central area of the SI basin to the east of Madagascar and from 15° to 30° S shows values of $H_{s}^{100} >$ 13 m (Fig. 3e). Values decrease towards the northwest coast of Australia with $H_{s}^{100}$ of approximately 12.7 m in this region. Similarly, values close to the coast of Africa are approximately 11 m.

These values are lower than any of the northern hemisphere basins. Although TCs in this basin have comparable values of $V_{max}$ (Fig. 2m) and frequency of occurrence (12.3/year) to the northern hemisphere basins^41^, the storms tend to track east to west and not frequently propagate to latitudes south of 35° S (see Fig. 2m, Fig. S2e). As a result, values of $V_{fm}$ are relatively low (Fig. 2o) in the areas of the most intense storms, explaining the smaller $H_{s}^{100}$ (Fig. 3e) compared to the northern hemisphere.

### South Pacific TC basin

The South Pacific tropical cyclone basin (SP) is bounded by the meridian of 240° E to the east and the east coast of Australia in the west (Fig. 2p–r). The largest value of $H_{s}^{100}$ calculated for the region was 14.7 m (Table 1) at 14.5° S; 189.5° E. Values of $H_{s}^{100} >$ 13 m (Fig. 3f) can be found in a longitudinal area from 160° to 200° E, including the Pacific islands of New Caledonia, Fiji, Tonga and Samoa. In the Coral Sea, (north-western area of the SP basin), $H_{s}^{100}$ varies between 11 m in the north part to 13.5 m in the south.

The climatology of the region is similar to the other southern hemisphere basin (SI). The values of $V_{max}$ tend to be smaller than the northern hemisphere (see Fig. 2^41^). Perhaps more importantly, however, as the southern hemisphere storms track more east–west they typically have smaller values of $V_{fm}$ (see Fig. 2r). When storms in this basin do move to higher latitudes, and $V_{fm}$ increases, they tend to have lower values of $V_{max}$ than in the northern hemisphere. As a result, values of $H_{s}^{100}$ are smaller than in the northern hemisphere and the maximum values tend to occur at the same latitudes as the maximum values of wind speed, $V_{max}$(see Figs. 2p, 3f).

## Conclusions

This study presents the first global-scale analysis of extreme-value (100-year return period) significant wave height across all TC basins. The results clearly show that the values of $H_{s}^{100}$ are influenced by both the maximum wind velocities and frequency of occurrence of storms in each basin. In addition, the largest values of $H_{s}^{100}$ do not necessarily correspond to the largest TC wind velocities, $V_{max}$. This occurs because within TCs, $H_{s}$ is not simply a function of the TC wind speed. In addition, the significant wave height depends on the velocity of forward movement of the storm, $V_{fm}$ and, to a lesser degree the spatial size of the storm.

As a result, in the WP and NA basins, where storm tracks propagate over a significant band of latitudes (recurving)^43^, storms move faster and become larger at high latitudes. This means that the largest values of $H_{s}^{100}$ occur at higher latitudes than the largest wind speeds. In contrast, in the EP and the southern hemisphere basins SI and SP, the TCs tend to track east–west over a limited range of latitudes and hence $V_{fm}$ does not vary greatly. Hence, the largest values of $H_{s}^{100}$ and $V_{max}$ tend to occur at similar latitudes.

The larger values of $V_{fm}$ and the greater frequency of occurrence of TC in the northern hemisphere means that values of $H_{s}^{100}$ for the northern hemisphere basins are larger than for the southern hemisphere.

The NI basin typically has relatively low values of $V_{max}$, although there are some extreme storms. The low frequency of occurrence of storms and the relatively small values of $V_{fm}$ means that the resulting $H_{s}^{100}$ are lower than either the WP or NA basins.

The results of the present study were compared with a wide range of previous studies for specific locations in each of the basins. Despite the wide range of data sources and analysis techniques adopted, our results are typically within $\pm$ 10% (see “Validation” section). To develop such a global analysis, however, numerous approximations and assumptions are necessary (see “Limitations” section). Source data for TC tracks and parameters, IBTrACS, is limited^40^. These data then form the basis for the synthetic STORM track database^41^. Finally, we apply a parametric wave prediction model^23^ and Extreme Value Analysis to the resulting significant wave height predictions. These steps obviously limit the accuracy of the final results.

## Methods

The aim of this study was to develop a two-dimension (latitude-longitude) distribution of $H_{s}^{100}$ for each of the global TC basins. In order to apply standard extreme value analysis (EVA) approaches^26^ it is necessary to develop a sufficiently long-duration estimate of extreme $H_{s}$ values at each location within these TC domains. The parametric TC model, PModel^23^ provides a computationally efficient method of generating such a significant wave height database. However, the model needs to be forced by wind field data for each basin covering a sufficiently long duration.

Initially, IBTrACS (Version 4) (International Best Track Archive for Climate Stewardship)^40^ data were used as a source for TC track information. Data from 1988 onwards were selected for this purpose, as many TC parameters are not available in earlier IBTrACS data^40^. A number of different EVA approaches were tested with this approximately 30-year duration dataset. However, it was concluded that there were insufficient data to form stable EVA estimates over the various TC basins.

To address this issue, it was decided to use a tropical cyclone synthetic database to generate the $H_{s}$ data for further analysis. The STORM (Synthetic Tropical cyclOne geneRation Model)^41^ database has been developed to have comparable track and wind field parameter statistics to IBTrACS data^40^. The STORM database contains a total of 10,000 years of synthetic TC tracks for each TC basin. For the present application the first 1000 years were selected for analysis in each TC basin. Bloemendaal et al.^41^ have compared the track distribution of the TC parameters with IBTrACS. The tracks of the 1000 year subset used for the present application are compared with IBTrACS in Bloemendaal et al.^41^ (their Fig. 4). In addition, comparisons with other TC wind field parameters are shown in the Supplementary Material and discussed below in the “Limitations” section.

The generation of the significant wave height field, over a period of 1000 years for each TC basin using a full 3rd generation spectral wave model (e.g. Wavewatch III)^44^ would be computationally prohibitive. Therefore, the parametric wave height model PModel^23^ was adopted. This model was developed based on Wavewatch III simulations over a wide range of TC conditions. For input, this model requires the TC parameters: track position, central pressure, $p_{0}$ or maximum wind velocity, $V_{max}$; radius of maximum winds, $R_{max}$; velocity of forward movement, $V_{fm}$ and radius to gales, $R_{34}$. With the exception of $R_{34}$ (see below), all these parameters are available or can be calculated from the STORM database. Note that IBTrACS uses a variety of averaging periods (1 min, 3 min, 10 min) to specify $V_{max}$. In the construction of the STORM database, these are harmonized to representative 10-min values^41^, consistent with the normal practice in wave modelling and as used in the development of the parametric wave model^23^.

**Figure 4:**
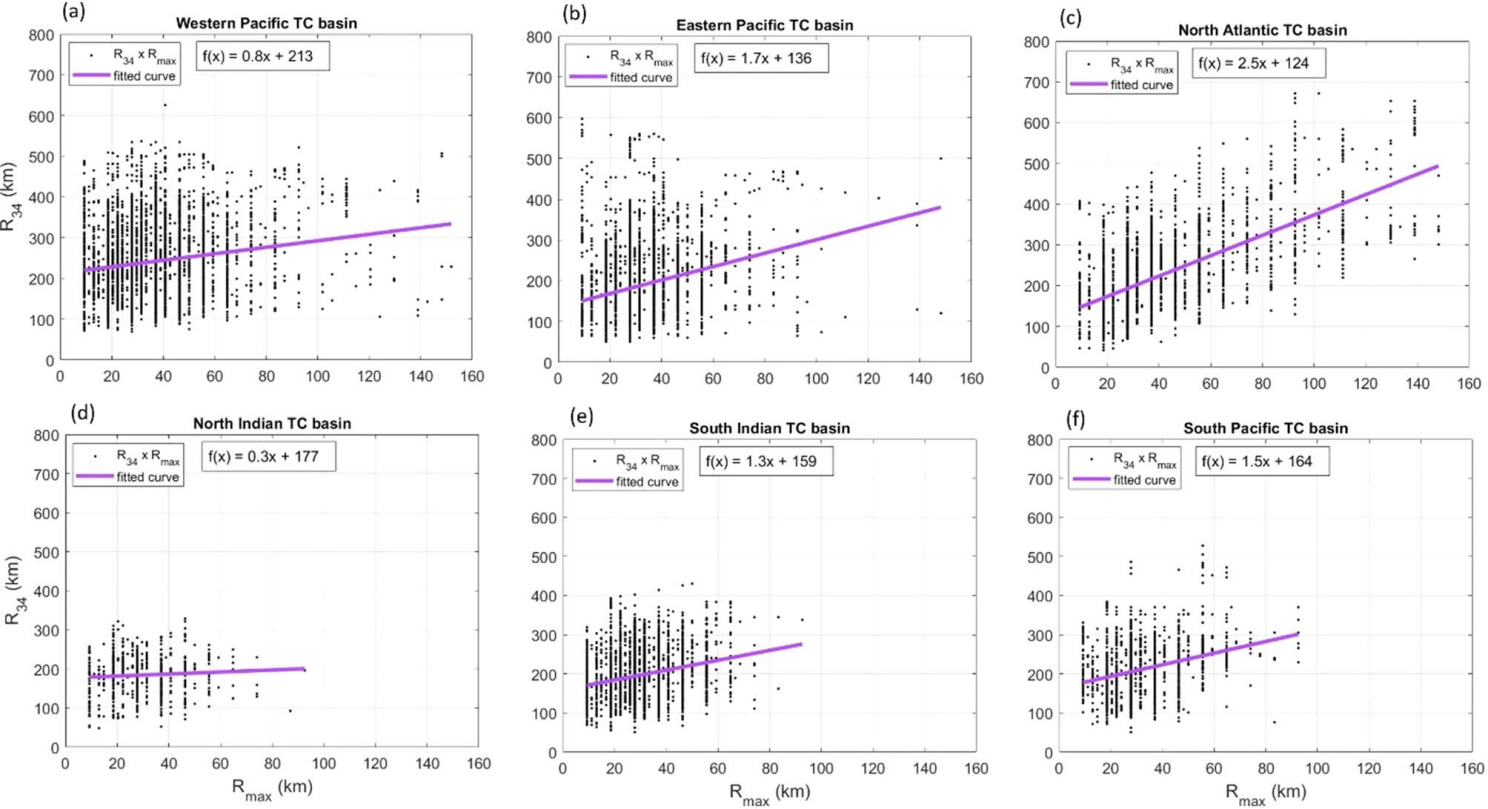
Relationship between $R_{34}$ and $R_{max}$, from IBTrACS data for each TC basin. The least squares curve fit to the data of Table 3 is shown by the solid line.

Following the definitions used in IBTrACS and STORM the six TC basins: Western Pacific (WP), Eastern Pacific (EP), North Atlantic (NA), North Indian (NI), South Indian (SI) and South Pacific (SP) were defined as in Table 1. The STORM database provides TC tracks for the full life cycle of the simulated storms, including when the TC transforms into an extra-tropical cyclone (ETC), at higher latitudes. In order to exclude the majority of ETCs, only STORM tracks within $\pm$ 45° of latitude were considered. In the meridional domain, the basin definitions were generally adopted as in the STORM database. The exception was for the EP, where the domain was truncated at 200° E, as the STORM wind intensities west of this limit appear unusually high compared with IBTrACS for the same region (see Fig. 4 of^41^).

Storms within the domains specified in Table 1 were selected from the STORM database if $V_{max} > 33$ m/s, the limit for a Category 1 TC on the Saffir-Simpson Hurricane Wind Scale. In addition, TCs were not considered once landfall occurred. The track position is defined within STORM on a three-hourly basis, with successive positions of the TC centre used to calculate the direction of TC propagation and $V_{fm}$. Figure 2 shows the resulting tracks, and values of $V_{max}$ and $V_{fm}$ for each domain.

### Values of radius to gales,$R_{34}$.

The STORM database does not provide information on the radius to gales, $R_{34}$. Fortunately, however, the resulting significant wave height field, predicted by the parametric wave model, is only weakly dependent on this parameter^23^. The options were to either adopt a constant (climatological) value of $R_{34}$ for each basin or an empirical relationship for this quantity. A number of studies have previously considered the relationship between $R_{34}$ on $R_{max}$^13,45–48^. This dependence was investigated for each basin using the IBTrACS dataset. Again, data within the domains specified in Table 1 and for which $V_{max} > 33$ m/s were selected. Figure 4 shows that there is a weak correlation between $R_{34}$ and $R_{max}$, although there is significant scatter. A least squares fit to the data was determined for each basin and is shown in Table 3 (all units are km). Note that alternative dependences on $p_{0}$ or latitude are also possible, as these quantities are linked.

**Table 3:** Empirical relationship between $R_{34}$ and $R_{max}$, from IBTrACS data for each TC basin.

Tropical cyclone basin	Least squares relationship (km)
Western Pacific	$R_{34} = 0.8R_{max} + 213$
Eastern Pacific	$R_{34} = 1.7R_{max} + 136$
North Atlantic	$R_{34} = 2.5R_{max} + 124$
North Indian	$R_{34} = 0.3R_{max} + 177$
South Indian	$R_{34} = 1.3R_{max} + 159$
South Pacific	$R_{34} = 1.5R_{max} + 164$

The larger spatial size of the northern hemisphere storms compared to the southern hemisphere is clear in Fig. 4. The one exception is the NI TC basin where the proximity to land may limit the size of TCs.

### Significant wave height database

The parametric significant wave height model, PModel^23^ was used to generate values of $H_{s}$ at each three-hourly time step for each synthetic TC across all six TC basins. The model defines values of $H_{s}$ within a region of 300 km from the centre of the TC. To ensure storm peaks were identifies, a grid with resolution 0.1° × 0.1° was defined for each TC basin and values of $H_{s}$ calculated at each grid point for each synthetic TC (1000 years of TCs for each basin). The maximum value of significant wave height, $H_{s}^{\max }$ was stored at each grid point during the passage of each TC (see Fig. 2).

### Extreme value analysis

In the present analysis, we aim to use extreme value analysis (EVA) to estimate the 1 in 100-year significant wave height, $H_{s}^{100}$ at each grid point of each TC basin. The value $H_{s}^{100}$ has a probability of occurrence, $P_{r} =$ 1/100 = 0.01 per annum^26^. In order to perform an EVA, the dataset must be independent, stationary and identically distributed^26^. That is, each observation must not be dependent on other observations, the mean statistics of the record shown should not vary with time (e.g. mean and standard deviation) and that each observation should follow the same probability distribution function. In EVA analysis, the time series is typically shorter than the desired return period (100-years in the present case) and a theoretical extreme value probability distribution is fitted to the data and extrapolated to the desired probability level (return period).

There are two common approaches used for EVA, block maxima and peaks over threshold^26,49^. The block maxima approach extracts annual maxima (AM) (or seasonal, monthly etc. maxima) from the data. Coles^26^ shows that such block maxim follow a Generalized Extreme Value (GEV) distribution. The use of annual values ensures that they are independent, but the approach can often be problematic as the use of annual values mean that there are typically not many data points to fit the GEV distribution (1 per year)^34^. A method commonly used to overcome this issue is to use peaks-over-threshold (POT), where data exceeding a given threshold are used. The peaks must be independent (e.g. not selected from the same storm). Such data follow a generalized pareto distribution (GPD)^26,49–51^. A significant issue in application of the POT approach is the selection of the threshold, where there is no theoretical guidance as to its value^24,33,52–55^.

In the present application, however, we have 1000 years of TC passes and hence there is no need to fit a PDF to the data and extrapolate to the required probability level. Rather, the $P_{r} =$ 0.01 level is “in sample” and can be calculated directly by rank-ordering of the data. This approach is called a direct return estimate (DRE)^28^. The DRE approach can be used with annual maxima values and, as there is no need to extrapolate a PDF, reduces statistical uncertainty.

For each synthetic storm in the STORM dataset, the maximum value, $H_{s}^{\max }$ generated by the parametric model was stored at the grid locations of the 0.1° × 0.1° grid. These data were pooled to form a 1.0° × 1.0° grid (i.e. 100 points were pooled). The annual maximum was then determined on this 1.0° × 1.0° grid. As there is 1000 years of synthetic storms, the number of annual maxima is 1000. The 1000 years of annual maxima were rank ordered from the largest to the smallest, with $H_{s}^{100}$ ranked at the 1000/100 = 10th data location^28^. However, in some years no TCs approach a grid square (see Figure S1). In these cases, the annual maximum from the TC population is zero but the total number of ranked values of annual maxima is still 1000. The number of non-zero data at each 1° × 1° bin is shown in Figure S1. This figure largely reflects the frequency of occurrence across each TC basin.

### Validation

As noted above, both the STORM TC track database and the parametric significant wave height model, PModel have been extensively validated in previous studies^23^. The aim here is to validate the predicted values of $H_{s}^{100}$. As this is a stochastic variable there is no “truth” to compare against. Rather, we must rely on independent EVA studies using a variety of statistical methods and data sources (e.g. buoy, satellite, model). The present analysis is global scale and, although our model is high resolution (0.1° grid), to address computational cost we have used a parametric model. This model has been extensively tested, is based on data generated with the Wavewatch III model and incorporates our present understanding of TC wave generation physics. However, it has limitations (see “Limitations” section). Therefore, point studies, which can use, for instance, a full 3rd generation spectral wave model (such as Wavewatch III) to generate a model dataset for subsequent EVA represents a useful validation source.

Table 4 shows a summary of a range of studies for each TC basin, with comparison values from the present study. Noting the broad range of data sources and methods applied, the agreement is reasonable. Generally, the present results are within $\pm$ 10% of the studies reported in the literature. Cases where there are differences larger than this magnitude can mostly be explained by known limitations in the present study methodology or the reported value from literature.

**Table 4:** Comparison of previous studies for each TC basin and the present study.

Basin	Location	Study type	Study $H_{s}^{100}$ (m)	Present $H_{s}^{100}$(m)
WP	South China Sea	Shao et al.^55^ 40 year model hindcast PoT/GPD EVA analysis	Max value 12.7 m	14.7 m See Fig. 3a
	North-west Pacific	Woo and Park^56^ 25 year satellite altimeter PoT EVA analysis	$\approx$ 16 m with max value 18 m	17.4 m See Fig. 3a
	Taiwan coast	Hsu et al.^57^ 20 year buoy records from 12 coastal locations (finite depth)	$\approx$ 12.3 m	14.5 m See Fig. 3a
	South China Sea	Du and Yan^58^ Spectra wave model with synthetic track data	Max value 24.4 m	19.8 m See Table 2 and Fig. 3a
	Vietnam	ISO oil industry design standards^59^ Various EVA approaches	10 m (12° N; 112° E)	12 m
			6.2 m (8° N; 106° E)	7 m
	Borneo	ISO oil industry design standards^59^ Various EVA approaches	7.0 m (8° N; 115° E)	6.9 m
	Philippines	ISO oil industry design standards^59^ Various EVA approaches	16 m	15.2 m
NA	GoM	Jonathan and Ewans^60^ Numerous buoy records Oil industry study (GOMOS—Gulf of Mexico Oceanographic Study) 315 stork peaks with PoT/GPD EVA analysis	$\approx$ 15 m	14–16 m See Fig. 3c
	GoM	Dentale et al.^61^ Spectral wave model WAM	17.9 m at buoy 42,040	14.9 m
			15.6 m at buoy 42,039	15.3 m See Fig. 3c
	GoM	ISO oil industry design standards^59^ Various EVA approaches	14.6 m	14-16 m See Fig. 3c
	GoM	API oil industry design standards^62^ Various EVA approaches	13.1 m (west)	12 m
			12.3 m (cent./west)	13 m
			15.8 m (central)	14-16 m
			12.2 m (east)	14-15 m See Fig. 3c
NI	Indian Ocean	Naseef and Kumar^63^ 39 years of ERA5^64^ global reanalysis data	12 m (NW Bay of Bengal)	15.5 m See Fig. 3d
SI	Indian Ocean	Naseef and Kumar^63^ 39 years of ERA5^64^ global reanalysis data	13.5 m (25.5° S; 79° E)	12.6 m See Fig. 3e
SP	Pacific between 15° and 25° S	Stochastic TC tracks Parametric wave height model^15^	13.2 m (210.4° E; 17.5° S)	12.8 m
			14.0 m (188.2° E; 13.8° S)	14.0 m
			12.5 m (190° E; 19.1° S)	13.4 m See Fig. 3f
	Coral Sea	Smith et al.^65^ 33 year altimeter data	9.6 m	10 m See Fig. 3f

Examples include the study of Hsu et al.^57^ for the coast of Taiwan where the buoys were in finite depth conditions and hence result in $H_{s}^{100}$ approximately 14% lower than the present analysis which is for deep water. The API design standards^62^ for the Eastern Gulf of Mexico specify a $H_{s}^{100}$ approximately 13% lower than the present study. This area will be protected by the coast of Florida which is not represented by the present PModel. The Bay of Bengal study of Naseef and Kumar^63^ uses ERA5^64^ data which will not adequately resolve TCs and hence produces $H_{s}^{100}$ estimates 23% lower than the present approach.

### Limitations

As with all analyses of extremes, the present approach has a number of limitations which should be considered. These limitations include elements associated with: (a) the STORM TC database, (b) the parametric significant wave height data and (c) the estimate of the statistical variable $H_{s}^{100}$.

#### STORM TC database

The STORM database^41^ has been developed so as to have similar statistical and track properties to the observational data of IBTrACS. Bloemendaal et al.^41^ show comparisons of STORM and IBTrACS mean quantities for each basin (their Fig. 5), including: TC genesis counts, minimum central pressure, maximum wind speed and radius of maximum winds. In the present application, our concern is focused on extremes rather than mean quantities. To test the similarity of STORM and IBTrACS datasets for extremes, Cumulative Distribution Functions (CDFs) for both datasets are shown in the Supplementary Material (Figure S2). The data were selected as used in the present application. That is, no data at latitudes greater than 45° in either hemisphere and only cases for which $V_{max} >$ 33 m/s were retained. CDFs for $p_{0}$, $R_{max}$ and $V_{fm}$ are shown. The results are generally consistent with the mean values given by Bloemendaal et al.^41^ with the two datasets in good agreement. In particular, it should be noted that for the SP basin, STORM has consistently higher values of $p_{0}$ than IBTrACS (by approximately 10HPa). This may result in a possible under-estimation of $H_{s}^{100}$ in this basin. Values of $R_{max}$ for the selected sub-set of the STORM database are larger than IBTrACS across all basins (by approximately 10 km). This may result in an over-estimation of $H_{s}^{100}$, although the dependence on $R_{max}$ is not as strong as for $p_{0}$(or $V_{max}$) and $V_{fm}$. Values of $V_{fm}$ are not directly available from STORM but were determined from successive track locations. As noted above, this is also an important parameter in defining maximum values of $H_{s}$. Therefore, it is reassuring that the comparison between STORM and IBTrACS for his parameter show excellent agreement (see Figure S2).

It is important to note that for TC wave generation, both $V_{fm}$ and $R_{max}$ play an important role in defining $H_{s}$, (in addition to $p_{0}$) as these quantities largely determine the “extended fetch” for a storm. Hence, errors in these quantities in the STORM database will flow through to the reliability of $H_{s}^{100}$. Understanding the role of the “extended fetch” continues to evolve as both the observational dataset^5,21^ and the theoretical underpinning^22,23^ grow. The model studies of Grossmann-Matheson et al.^23^ have reinforced the important role of non-linear interactions in TC wave generation. One of the consequences of this is that wave growth can be sustained for cases where waves are propagation faster than the local wind speed. This effectively means that the “extended fetch” is larger than previously believed (this new understanding is incorporated in the parametric model used here) and the dependence on $V_{fm}$ and $R_{max}$ is less dramatic (“detuned”) than assumed in the past. In the present context, this means that errors in $V_{fm}$ and $R_{max}$ are less important than previously believed. Nevertheless, the role of these quantities is still significant and enhancements in the observational datasets (IBTrACS) and derived synthetic databases, such as STORM, will be important for future studies.

#### Parametric significant wave height database

The parametric significant wave height model was extensively validated against buoy and satellite altimeter data by Grossmann-Matheson et al.^23^. Although the model generally performs well, it was developed based on a computational dataset for TCs moving along a uni-directional track and in deep water. Therefore, the model can be expected to overestimate in cases where the TC wind field parameters change rapidly or the track has high curvature. In addition, as the model is deep water, it will generally over-estimate significant wave height in finite depth condition and where land may limit wave growth.

#### Statistical variability of $H_{s}^{100}$

In conventional EVA analysis, where a PDF is fitted to the data and extrapolated to the desired extreme value probability level, the goodness of fit to the data can be used to estimate confidence limits for the extreme value^26^. In the present application, we have a dataset of 1000 years and hence it is not necessary to fit a PDF to the data and extrapolate to the 1 in 100-year event. The values of $H_{s}^{100}$ can be estimated from the rank-ordered data using the DRE method^28^. Although this greatly reduces the statistical variability in the resulting estimate, confidence is still limited by the ability to accurately define the tail of the distribution. In the present case, this is defined by the reproducibility of the ranked extremes. The 95% confidence limits on the DRE estimates of $H_{s}^{100}$ were investigated using a bootstrap approach^28,35,66^. As shown by Breivik and Aarnes^66^, estimation of EVA tail statistics can be obtained by using only a sub-set of the k-highest values in a DRE. Following this approach, at each 1° grid point, the top 100 values in the DRE were selected. These values were randomly sampled with replacement to generate new ranked series, from each of which the 10th point was selected to form an estimate of $H_{s}^{100}$. This process was repeated 500 times and the values of $H_{s}^{100}$ used to estimate the 2.5% and 97.5% values. The difference between these values defines the estimate of the span of the 95% confidence interval ($CI_{95\% } = H_{s}^{100} (97.5\% ) - H_{s}^{100} (2.5\% )$). To test the validity of the approach, the full datasets were also used (rather than the top 100 values). The results were almost identical.

Figure S3 shows the values of $CI_{95\% }$ for each of the TC basins. These results show that the confidence interval is typically between 1.0 and 1.5 m for most areas. In regions where the frequency of storms is lower (Figure S1), the values of confidence limits increase (up to 3 m). These areas of reduced statistical confidence include: the Gulf of Mexico, the higher latitudes of the EP and SI basins, the lower latitudes of the SP and WP basins and all of the NI basin. As values of $H_{s}^{100}$ are typically 15 m for most basins, this means the confidence interval is generally approximately 10% (1.5/15) or ($\pm$ 5%). Thus the DRE results in relatively small statistical variability. Note, as pointed out above, there is, however, additional variability due to the accuracy of the synthetic track dataset and the parametric wave model.

### Supplementary Information

Supplementary Information.

## Data Availability

The spatial distributions of $H_{s}^{100}$ for each of the TC domains on 1° × 1° grid are available at 10.26188/24448276.v2.
