# Supplementary material for: Global tropical cyclone extreme wave height climatology
Source: Sci Rep. 2024 Feb 20;14:4167. doi: 10.1038/s41598-024-54691-9 (PMC10879538; doi:10.1038/s41598-024-54691-9)
Supplement: Supplementary file 1 — Supplementary Information. [file 41598_2024_54691_MOESM1_ESM.pdf]

Supplementary Material for:

Global Tropical Cyclone Extreme Wave Height Climatology

Guisela Grossmann-Matheson[1], Ian R. Young[1*], Alberto Meucci[1], Jose-Henrique Alves[2]

[1]Department of Infrastructure Engineering, University of Melbourne, Melbourne, Australia
[2] Weather Program Office, Ocean and Atmospheric Research, NOAA, Silver Spring, MD, USA
* Corresponding author, ian.young@unimelb.edu.au

This PDF file includes:

Figs. S1 to S3

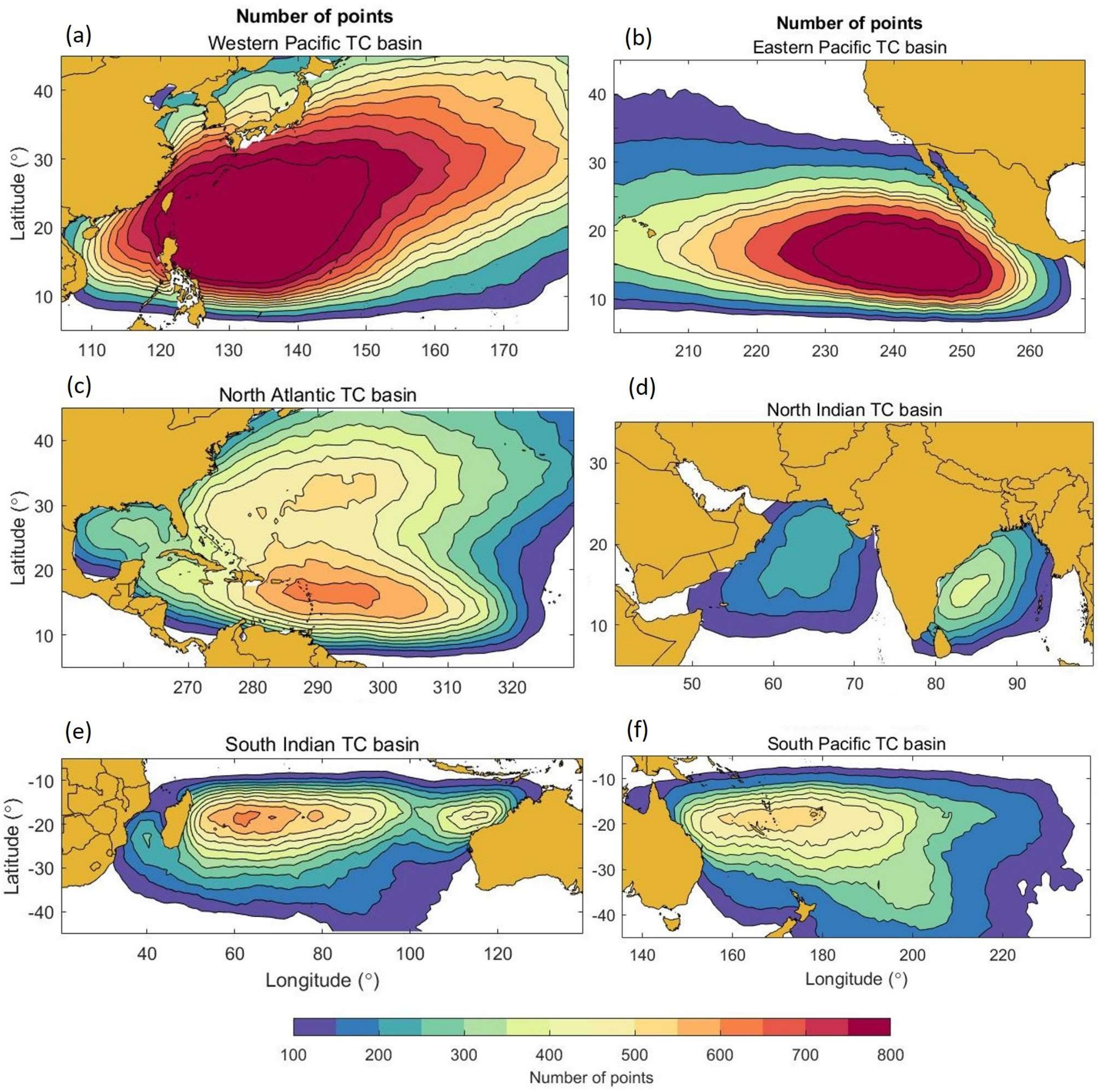

Figure S1. Contour plots of the number of non-zero annual maxima values of H_s at each 1°x1° grid location used in the extreme value analysis to determine the 100-year return period extreme significant wave height. (Figure created with Matlab R2023a – mathworks.com)

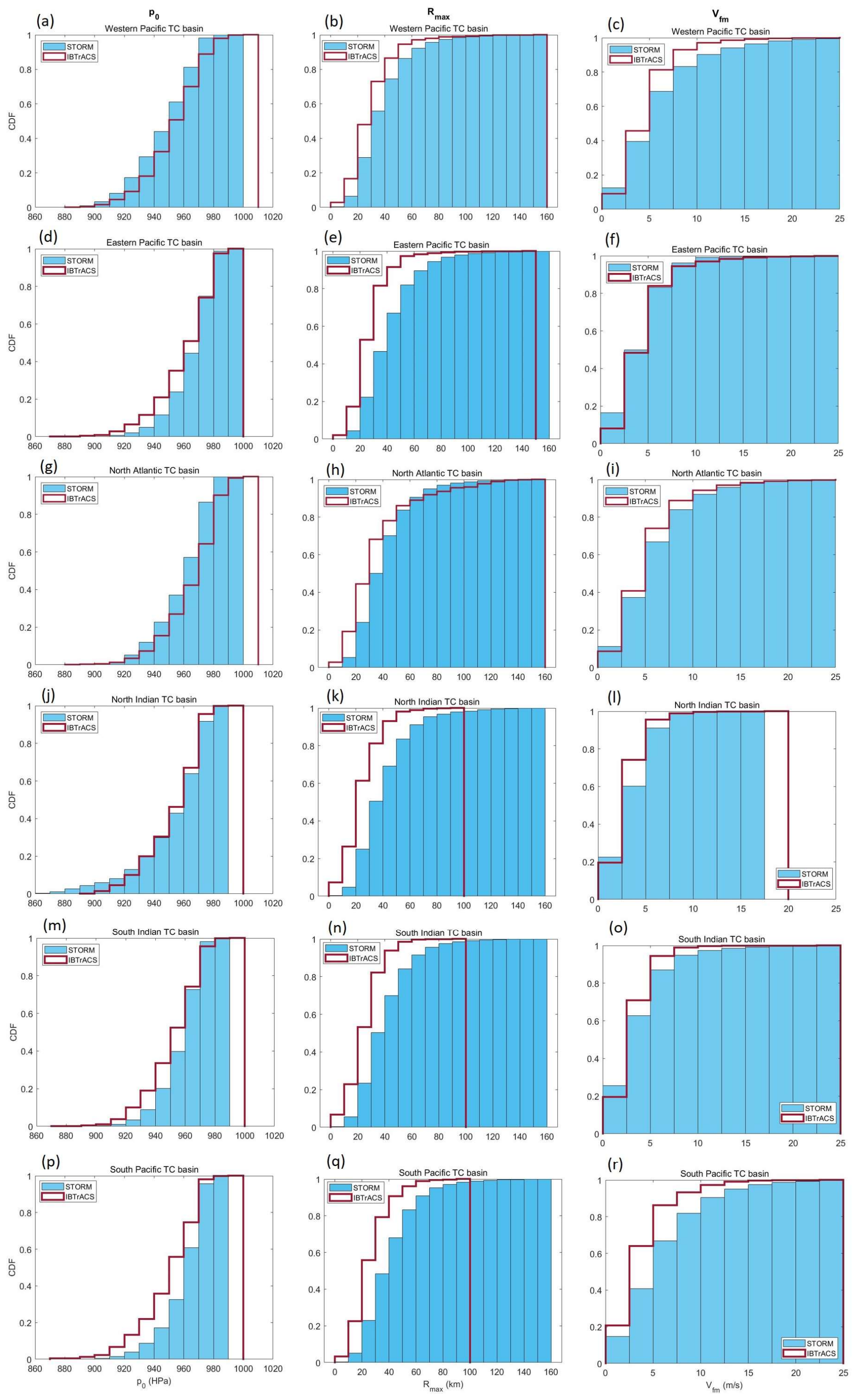

Figure S2. Comparison of the cumulative distribution functions for TC parameters from IBTrACS and the subset of STORM used for the present analysis for each TC basin. Column 1, central pressure, p_0 ; Column 2, radius to maximum winds, R_{max} ; Column 3, velocity of forward movement, V_{fm} .

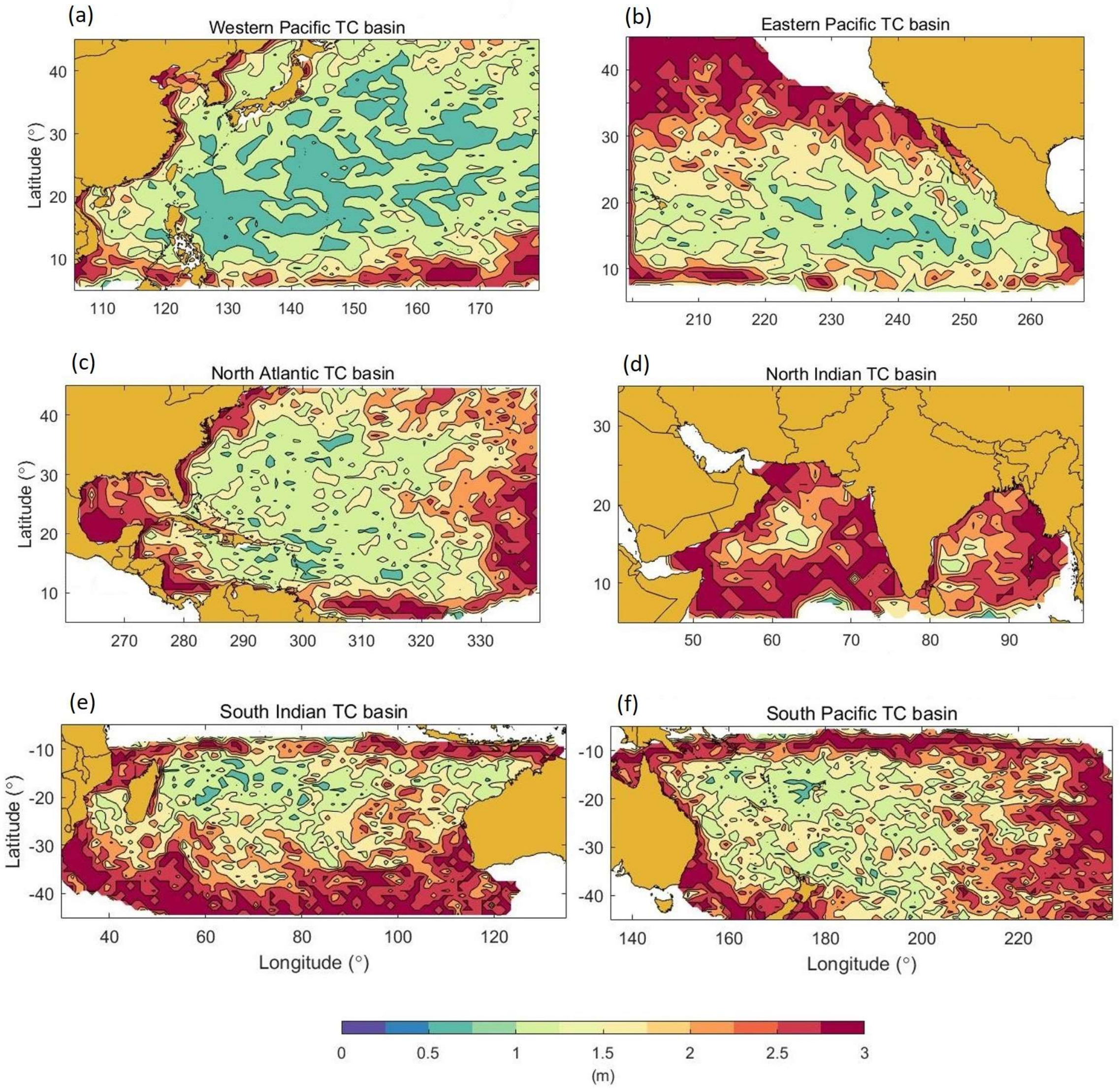

Figure S3. The 95% confidence interval on the 100-year significant wave height for each TC basin. Determined by a bootstrap approach with the 100 largest values at each 1°x1° grid location. The confidence interval is the difference between the 97.5% H_s^{100} minus the 2.5% H_s^{100}. (Figure created with Matlab R2023a – mathworks.com)
